# The development and evaluation of content validity of the Zambia Spina Bifida Functional Measure: Preliminary studies

**DOI:** 10.4102/ajod.v6i0.264

**Published:** 2017-07-24

**Authors:** Margaret M. Mweshi, Seyi L. Amosun, Mary P. Shilalukey-Ngoma, Esther Munalula-Nkandu, Zuhayr Kafaar

**Affiliations:** 1 Department of Physiotherapy, School of Medicine, University of Zambia, Zambia; 2 Division of Physiotherapy, School of Health & Rehabilitation Sciences, University of Cape Town, South Africa; 3 Department of Paediatric & Child Health, School of Medicine, University of Zambia, Zambia; 4 Department of Psychology, Faculty of Arts & Social Sciences, Stellenbosch University, South Africa

## Abstract

**Background:**

Very little is known on outcome measures for children with spina bifida (SB) in Zambia. If rehabilitation professionals managing children with SB in Zambia and other parts of sub-Saharan Africa are to instigate measuring outcomes routinely, a tool has to be made available. The main objective of this study was to develop an appropriate and culturally sensitive instrument for evaluating the impact of the interventions on children with SB in Zambia.

**Methods:**

A mixed design method was used for the study. Domains were identified retrospectively and confirmation was done through a systematic review study. Items were generated through semi-structured interviews and focus group discussions. Qualitative data were downloaded, translated into English, transcribed verbatim and presented. These were then placed into categories of the main domains of care deductively through the process of manifest content analysis. Descriptive statistics, alpha coefficient and index of content validity were calculated using SPSS.

**Results:**

Self-care, mobility and social function were identified as main domains, while participation and communication were sub-domains. A total of 100 statements were generated and 78 items were selected deductively. An alpha coefficient of 0.98 was computed and experts judged the items.

**Conclusions:**

The new functional measure with an acceptable level of content validity titled Zambia Spina Bifida Functional Measure (ZSBFM) was developed. It was designed to evaluate effectiveness of interventions given to children with SB from the age of 6 months to 5 years. Psychometric properties of reliability and construct validity were tested and are reported in another study.

## Introduction

Spina Bifida (SB) is one of the congenital malformations of the central nervous system that is a major and unrecognised expensive public health problem in much of Africa (Adeleye, Magbagbeola & Olowookere 2010; Blenchowe et al. 2010; Mweshi et al. 2015). It is the commonest of the neural tube defects, and hydrocephalus commonly occurs in association (Fabiano, Doyle & Grand 2010; Qureshi 2010; Sacko et al. 2010). The two are the most recurrent and disabling malformations in neonates in the sub-Saharan African paediatric environment which have a huge impact on the functioning of a growing child (Mweshi et al. 2010).

Children with SB need specialists who can address problems related to hydrocephalus, neurogenic bowel and bladder, mobility, learning disabilities and functional limitations. They also require generalists who can help educate caregivers and address health promotion issues, including nutrition and exercise. Thus, a multidisciplinary team comprising neurosurgeons, neurologists, orthopaedic surgeons, urologists, physiotherapists, paediatricians, neuro-nurses, rehabilitation specialists, psychologists and social workers is what is recommended for the management of children with SB (Mitchell et al. 2004). Consequently, the delivery of this complex care requires an integrated system that aligns and informs all parties involved (Adzick et al. 2011; Liptak & El Samra 2010).

Studies performed on the management of children with SB in some African countries such as Nigeria, Cameroon, Kenya, Uganda and Zambia have reported challenges encountered in the management of SB (Adeleye et al. 2010; Blenchowe et al. 2010; Mweshi et al. 2015). No outcomes have been reported on the management of children with SB in sub-Saharan Africa; hence, knowledge on instrument measures that could be used has been unavailable. Given the several studies performed in many African countries without reported evidence of the impact of management, one could probably assume that either appropriate instrument measures are inaccessible or that they do not just exist. This situation ultimately creates a gap in the provision of evidence of the impact of interventions given to such children in the regions of sub-Saharan Africa. Therefore, in order to investigate how other rehabilitation professionals outside the region manage to measure the impact of the interventions given to children with SB, a systematic review was carried out.

The search strategies used were the Cochrane, Database Specification Review, Autodesk Certified Professional Journal Club, Database of Abstract Reviews Effects, Cochrane Controlled Trial Register, Comprehensive Microbial Resource, Health Technology Assessment and National Health Service Economic Evaluation Database from 1950 to January 2010. A total of 705 (*n* = 705) titles and abstracts related to the topic were retrieved and reviewed. Eighty-two (*n* = 82) titles were deemed relevant by the researchers. Subsequently, data were extracted from all fitting methodological articles (*n* = 19) of which six (*n* = 6) were located and critiqued. Consequently, four (*n* = 4) studies were critically appraised and evidence was reported.

The results of the search showed that the instruments identified were the Gross Motor Function Measure (GMFM) Dimensions D and E, Pediatric Outcomes Data Collection Instrument Parent and Child versions, Gillette Functional Assessment Questionnaire Walking subscale, Functional Independence Measure for Children (WeeFIM), Pediatric Quality of Life Inventory, temporal–spatial gait parameters, O(2) cost during ambulation, Child Health Questionnaire, Functional Mobility Scale, Pediatric Evaluation of Disability Inventory (PEDI), CP QOL-Child, and QOL (KIDSCREEN), Bruininks-Osserestsky tests, Alberta Infant Motor Scale and Bayley Scale of Infant development (Harvey et al. 2008; Oeffinger et al. 2007; Sullivan et al. 2007). Subsequently, the search revealed 11 outcome measures of which two are commonly used tools for measuring interventional outcomes in children: the PEDI and the WeeFIM validated for American children (Berg et al. 2008; Sirzai et al. 2008; Sonel et al. 2009).

Based on the results of the literature search, it can be concluded that there is no empirical data showing evidence of the PEDI and WeeFIM being translated into any of the African languages and their usage in Africa. However, although the two measures have not been so easily available and perhaps applicable for Zambian children, there is a lot that could be learnt from the same measures. On the other hand, it is also extremely important to note that there has been a paradigm shift of thinking from a developmental focus to functional focus in paediatric rehabilitation. For instance, worldwide researchers and clinicians who have used the PEDI have highlighted variations in functional skill acquisition in clinical populations. Furthermore, the importance of recognising cultural differences and the value of documenting functional progress in relation to interventions must be upheld (Haley et al. 2010). It is therefore quite imperative to recognise the shift of thought from the original authors of the PEDI who at one time encouraged the idea of translating the tool into local languages while using the normative data from the USA to determine whether a deficit or delay existed with regard to functional skill development (Berg et al. 2008).

Additionally, there has been some debate over issues of culture and the importance of cultural validation of norm-referenced tests (Berg et al. 2008; Sirzai et al. 2008; Sonel et al. 2009). Despite the consensus on what appears to be the impact of culture on the functioning of children, some efforts have been made to translate the PEDI into Dutch, Norwegian, Swedish, Spanish, Turkish, Portuguese, Slovene, Icelandic, French, Hebrew, Japanese and Chinese languages (Haley et al. 2010; Jahnsen et al. 2002). A number of these international users have reported challenges applying the PEDI to their own culture. One of the issues in translating the PEDI is finding comparable words in each country’s language. For example, a Norwegian team has reported difficulty finding comparable Norwegian words for ‘prompting’, ‘fasteners’ and ‘item’. Cultural differences required item adaptations and additions to the PEDI, for example the Dutch team added ‘bicycling’ to their mobility scale.

Inasmuch as facilitating international comparison is extremely essential in some cases, comparing the lifestyle of an American child with a typical Zambian child in terms of function may not be easily justifiable. This is because ethno-theories of most countries in the developed world are very different from those of the developing world because of cultural diversity. For example, Zambian children start crying for food at a later stage compared with Dutch and Turkish children (Willemsen & Fons 1997). This just highlights the importance of recognising the ability of a growing Zambian child to communicate the need to eat and drink because children are breastfed for a very long time and may need to develop the survival skills after being weaned from breast milk. This is supported by the notion that breastfeeding in Zambia is on demand and when the child is no longer breastfed there is a separation from the mother physically and emotionally (Chibuye, Mwenda & Osborne 1986). Other differences in culture for instance are that the PEDI and WeeFIM include the use of fork and knife in the evaluation. The two utensils may be considered unsafe for use by Zambian parents or caregivers of children with disabilities. It is therefore clear that instruments such as the PEDI or WeeFIM that are in use in the USA and Europe may lack appropriate items essential for Zambian children and may also include tasks and materials which are not encouraged in the Zambian culture. Therefore, the two instruments may not be easily applicable on Zambian children. As a consequence, Clinicians like physiotherapists managing children with SB in Zambia, cannot effectively quantify the impact of interventions given to the children and hence cannot produce evidence (Mweshi et al. 2011:20).

As evidence-based practice (EBP) and initiatives to improve the quality of healthcare and life in children with disabilities have grown around the world, recognition of the need to measure functional outcomes in all healthcare settings has also increased. While there has been such increasing emphasis on the provision of evidence by rehabilitation professionals worldwide (Kaplan 2007), rehabilitation outcomes have been less reported in the developing world because of limited and lack of appropriate instrument measures. The inability of appropriate measures should provoke African researchers to be innovative and develop measures that are culturally sensitive to the needs of African children with disabilities.

Considering the lack of specific outcome measures developed for evaluating the impact of interventions given to children with SB and lack of appropriate and culturally sensitive tools among the ones available, it was deemed necessary that a measure be developed in order to measure the level of functioning in children with SB in Zambia. In view of such limitations and the relevance of using a psychometrically sound instrument in paediatric rehabilitation, we set out to develop a culturally appropriate, multidisciplinary and sensitive functional measure for children with SB in Zambia and subsequently tested it for psychometric properties. The purpose of this paper was to describe the processes involved in the preliminary development and content validation of the Zambia Spina Bifida Functional Measure (ZSBFM).

## Methodology

The study was carried out at the University Teaching Hospital (UTH) and Beit Cure Hospital (BCH). Both the hospitals, which are the only centres providing specialised care to children with SB in Zambia, are found in Lusaka. The two hospitals were comprehensively informed of the nature of the study through letters of permission. The initial process of instrument development involved the identification of the main domains of care in children with SB through a nine-year retrospective study, while confirmation of domains was done through a systematic review of literature. Eventually, parents and caregivers of children with SB and youths with SB were recruited to participate in the process of item generation. Subsequently, expert clinicians managing children with SB validated the items, and ultimately the measure called ZSBFM was constructed. In total, four studies were carried out in the whole process of instrument development.

The methodology section comprises the mechanisms used to identify study participants, followed by the procedures that were undertaken to collect data. Eventually, methods of data analysis used in the studies will be presented.

### Identification of participants for the studies

Table 1 presents samples for all the four studies involved in the initial development of the ZSBFM. Study 1 conveniently identified children with SB and hydrocephalus from whom domains of care were identified. Study 2 captured external data of the appraised studies in the systematic review process. In Study 3, purposive samples were used including participants with experience of caring for children with SB and other participants who were youths with SB. Eventually, clinicians were purposively identified for Study 4 from the UTH and BCH for the content validation exercise, and subsequently three content specialists, being two physiotherapists and one nurse, were also conveniently identified for the item–objective congruence exercise. Table 2 shows the demographic details of the expert panel.

**TABLE 1:** Study samples for the studies involved in the development of the Zambia Spina Bifida Functional Measure.

Study 1: Retrospective study	Study 2: Systematic review	Study 3: Item generation study	Study 4: Content validity study and item–objective congruence evaluation
One thousand four hundred children with SB and hydrocephalus (*n* = 1400)	Appraised studies (external data) - Study A (*n* = 55) - Study B (*n* = 34) - Study C (*n* = 151) - Study D (*n* = 895)	- Twenty youths with SB (*n* = 20) - Twenty parents/caregivers of children with SB (*n* = 20)	- Twelve clinicians (*n* = 12: content validity study) - Three clinicians (*n* = 3: item–objective congruence evaluation)

*Source*: Authors’ own work

**TABLE 2:** Demographic details of the expert panel.

Expert identity	Profession/role	Highest qualification	Years of experience
A	Physiotherapist‡	Diploma	13 years
B	Physiotherapist‡	Bachelor’s	15 years
C	Physiotherapist‡	Diploma	28 years
D	Neuro-nurse‡	Diploma	16 years
E	Neuro-nurse‡	Diploma	11 years
F	Neuro-nurse‡	Diploma	28 years
G	Clinical officer‡	Licentiate	15 years
H	Physiotherapist†	Master’s	32 years
I	Physiotherapist‡	Master’s	35 years
J	Clinical officer‡	Diploma	40 years
K	Neuropediatrician‡	Master’s	15 years
L	Neurosurgeon‡	Master’s	20 years

*Source*: Authors’ own work

† , Academic;

‡ , Clinician.

### Procedure of data collection

The procedures involved in the process of data collection and final instrument construction will be presented in four sections:

- Domain identification
- Domain confirmation
- Instrument preparation
- Item generation, content validation and item–objective congruence evaluation

#### Domain identification

The process of identifying the domains of care started by orientating three research assistants who are physiotherapists by profession. They were oriented on how to extract relevant information from the clinical files using a data-capturing sheet and eventually entering data into the SPSS database. Upon receiving ethical approval, permission from the hospital administrators of the two hospitals was sought. A checklist was then adapted from the assessment form routinely used for children with SB at the BCH. The viability of the checklist was tested by piloting and subsequently validated by three physiotherapists, three neuro-nurses, one orthopaedic surgeon and two neurosurgeons. Upon validating the checklist, domains were identified from the clinical files of children with SB and hydrocephalus identified from 2001 to 2010 (Mweshi et al. 2011).

#### Domain confirmation

To confirm the domains of care that were identified, a systematic review study was performed. The clinical question was:

What is the evidence that the functional domains of self-care, mobility, social function, participation and communication can be used to measure function in children with SB following an intervention in Zambia?

A critical appraisal of functional outcomes studies and commonly used functional outcome measures with their psychometric properties in measuring the impact of interventions was performed. This whole process was based on external data from four studies giving a sample size of 1135 participants (Table 1: Study 2).

#### Instrument preparation

Preparation for instrument development is essential before items are generated. Therefore, it becomes necessary to identify methods of administration, number of items testing each objective or subscale, item formats and test scoring in the preparation of instrument specifications.

#### Method of administration

The instrument is expected to be administered by clinicians with the help of primary caregivers, based upon their direct observations of the child’s behaviour in performing functional activities. To facilitate a multidisciplinary approach which is needed for SB management, the ZSBFM has been principally designed for use by physiotherapists, occupational therapists, neuro-nurses, neurosurgeons, orthopaedic surgeons and clinical officers in Zambia. It is expected to provide an examiner’s guide and a summary scoring form, with graph paper.

#### Number of items testing each objective

The establishment of the number of items began by a process of blueprint development. This was initiated by formulating a set of objectives reflecting the outcomes and critical areas to be assessed. Below is a list of objectives that were set:

- to determine the levels of performance of self-care, mobility and social function in children with SB in their activities of daily living,
- to ascertain the ability of children with SB to communicate the functional needs in performing activities of daily living,
- to ascertain the ability of children with SB to participate in performing functional activities.

The next strategy was concerned with the total number of items that would make up the ZSBFM. Based on the numbers of items in commonly used measures such as the WeeFIM with 18 items, BDI with 61, GMFM with 88 and the PEDI with 241 items, the researchers made a resolve to develop a measure that would neither be too short nor too long.

The major content areas to be assessed included self-care, mobility and social function that appeared as column headings across the top of the table and critical areas assessed being communication and participation that appeared on the left side as row headings. At each intersection was a particular content-objective pairing and values in each cell reflecting the actual numbers of each item that were to be included in the proposed draft measuring instrument. The range of the number of items picked by the researchers was between 70 and 80. It was suggested that the total number of items for the three main domains would be between 50 and 60 items, while items on the sub-domains would be between 10 and 15 items each.

A total of 52 items were suggested to reflect the three main domains of which 26 items were earmarked for self-care, 18 items for mobility and 8 items for social function. With regard to communication, a total of 13 items were proposed, of which 5 items represented communication in self-care and 8 items communication in social function, while none was suggested for the domain of mobility. Participation was equally given a proportion of 13 items of which 5 items reflected participation in self-care, 3 items represented participation in mobility and 5 items were earmarked for participation in social function. Table 3 shows the blueprint that was ultimately constructed in the preparation of the test specifications showing the number of proportions and items that were subsequently generated.

**TABLE 3:** Blue print showing the number of portions and items that were proposed for developing the measure.

Objectives	Content of functional skills			

	Self-care	Mobility	Social function	Total
To determine the levels of performance of self-care, mobility and social function in children with SB in their activities of daily living	26	18	8	**52**
To ascertain the ability of children with SB to communicate the functional needs in performing activities of daily living	5	0	8	**13**
To ascertain the ability of children with SB to participate in performing functional activities	5	3	5	**13**

**Total**	**36**	**21**	**21**	**78**

*Source*: Authors’ own work

#### Identification of the scoring rules and procedures

There are basically four classic scales or levels of measurement presented in literature being nominal, ordinal, interval and ratio scales. Well-renowned measures such as the GMFM 88 have utilised the scale in the use of the four-point ordinal scale (Avery et al. 2003; Russell et al. 1989). Given the potential advantages of using such a scale, the current study adopted a four-point Likert scale (1–4). The researchers adopted a model that awards scores for performing a functional task from 4 to 1, with each statement giving equal weighting as it has been suggested that differential weighting brings about potential problems of calculation (Avery et al. 2003; Bjornson et al. 1998; Russell et al. 1989). The final score is expected to be obtained by summing individual items. Nonetheless, the expected final scores for the age ranges of 6 months to less than 2 years, 2 years to less than 3 years and 3 years to less than 5 years are different because some of the functional skills are age dependent. The results of a total score of a domain can be interpreted that a child has 100% probability of having a score of 4 on every item of a domain.

#### General instructions for awarding scores for the performance of the task

The items of functional skills of children aged 6 months to 5 years are arranged into three sections. Section one has items on self-care, followed by the section on mobility and lastly social function. Instructions state: Please indicate by ticking (√) the statement that best describes the child’s ability to perform each of the following activities taking into consideration the appropriate age category. Please note that blocked spaces in the age categories of 6 months to 2 years and 2 years to 3 years show that the child is young for the activity in question. However, the scores to be awarded are from a range of 4 to 1, with the following interpretations:

*Score 4*, independent of caregiver, can perform the activity with or without mechanical aids

*Score 3*, independent of caregiver, but needs monitoring or aid in performance of activity

*Score 2*, requires assistance by caregiver or mechanical aid in performance of activity

*Score 1*, completely dependent, needs help with activity.

#### Item generation process, content validity and item–objective congruence evaluation

Upon identifying and confirming the domains of care and formulating the specific instrument preparation guide, the researchers immediately went into specific item generation. This process was followed by the process of preliminary item validation and, subsequently, the congruence of the items was evaluated.

#### Process of item generation

The process of item generation involved the qualitative enquiry of semi-structured interviews and focus group discussions (FGDs). A summary of questions asked in the interviews and focus groups is presented in Appendix 1. For the purpose of congruent items, themes and question guides from both interviews and FGDs were generated from the blueprint and are shown in Table 3. As soon as everything was put in place, a pilot study was performed to ensure that items would be extracted from the two methods of enquiry. Semi-structured interviews were conducted before the FGDs in order to identify relatively personal views before validating the general consensus views.

- Semi-structured interviews
  ■ A total of 20 semi-structured interviews were conducted in the study. Appointments with the research participants were made during the clinics at both hospitals. All the interviews were carried out at Cheshire Homes Rehabilitation Centre for children with disabilities. Before interviews started, informed consent was obtained from all participants and permission to record interviews was sought. Participants were asked what language they were comfortable with, and the main researcher identified a research assistant in instances where she was not so comfortable with the preferred language of the participant. Confidentiality was ensured and the participants were made comfortable by creating an atmosphere that facilitated freedom of expression.
  ■ The first five interviews were conducted with youths and the next five with parents, or caregivers, followed by five youths and then the last five parents, or caregivers, giving a total of 20 interviews. Codes were given to the participants in order to facilitate easy analysis. Codes A1–A10 were given to youths who took part in the semi-structured interviews while B1–B10 to mothers or caregivers. For the purpose of quality listening, a maximum of three interviews were conducted in a day. This was meant to create ample time for the researcher to download the recorded interviews and transcribe them with ease. On average, interviews took between 45 minutes and 1 hour 30 minutes.
- Focus group discussions
  ■ Upon getting consent from parents, or caregivers, and assent from the youths with SB, dates and times for the two FGDs were set. The first FGD comprised youths with SB (*n* = 10) while the second was with parents or caregivers (*n* = 10) of children with SB. Codes C1–C10 were given to youths, while D1–D10 to mothers or caregivers in order to facilitate easy management of data. The two FGDs took place at Cheshire Homes for Children with Disabilities in Kabulonga, and confidentiality was ensured before commencing the FGDs.

#### Content validation and item–objective congruence evaluation

When investigating content validity, the interest is in the extent to which the measure represents the content domain (Waltz, Strickland & Lenz 2010). At least two or three experts in the area of the content to be measured can evaluate the validity of the items. When only two or three judges are employed, content validity index (CVI) is used to measure the level of agreement between the experts. When more than two or three experts rate the items on a measure, the alpha coefficient is employed as the index of content validity. Therefore, in order to be more inclusive, a resolve was made to involve 12 different clinicians who are involved in the management of children with SB and 3 for the item–objective congruence evaluation.

In order to validate the items generated from the interviews and FGDs, appointments with 12 expert clinicians were arranged in person to explain the purpose of the evaluation. Letters explaining the aim, the purpose of the questionnaire and procedure of administration were given to each research participant. Subsequently, the experts were given the objectives of the measure and a list of generated items. They were asked to independently rate the relevance of each item using a 4-point rating scale: 1 not relevant, 2 somewhat relevant, 3 quite relevant and 4 very relevant.

## Methods of data analysis

### Qualitative analysis

Of paramount importance to data quality is the accuracy of the transcribed interviews and FGD notes (Waltz et al. 2010). Given the purpose of the study and the type of data collected, the choice of type of analysis was manifest content analysis. Therefore, the analysis of both semi-structured interviews and FGDs involved downloading of recorded data, translation into English and transcribed verbatim data were then placed into categories of the main domains of care deductively. The results of both the interview and focus group methodologies were categorised under similar themes and finally the back and forth potential verification with some of the original information helped to strengthen the analysis.

### Quantitative analysis

Descriptive statistics were used to analyse quantitative data by using SPSS version 17. The level of statistical significance was set at *p* ≤ *0.05* at 95% confidence interval. Internal consistency was measured by Cronbach’s alpha. Validity was measured by using both Item Content Validity Indices (I-CVIs) and Scale Content Validity Indices (S-CVIs) (Waltz et al. 2010).

### Instrument construction process

The process of instrument construction involved compiling all the necessary components essential for the instrument measure. It involved designing the cover page presenting the title of the tool and the age limit for using the tool and the name of the instrument developer. Also found on the cover page is a provision for brief information about the interviewer, respondent and about the child concerning information on SB and services such as surgery, orthotics and physiotherapy and general instructions on the use. General instructions on awarding scores for the testing different functional skills to facilitate uniformity in assessing the levels of function in the children were also put in place. The items of functional skills of a child aged 6 months to 5 years are arranged into three sections with items on self-care, followed by the section on mobility and lastly social function. Instructions state: Please indicate by ticking (√) the statement that best describes the child’s ability to perform each of the following activities taking into consideration the appropriate age category. Please note that blocked spaces in the age categories of 6 months to 2 years and 2 years to 3 years show that the child is young for the activity in question. Lastly, the summary scoring form that provides the clinician with raw scores for each sub-section and also a graph for plotting in order to monitor if there is progress or no progress in the management programme was also compiled.

## Results

The results section presents the domains identified and confirmed, items generated from qualitative data, results of the content validation exercise and the item–objective congruence exercise. Subsequently, the process of instrument construction will be presented.

### Domain identification and confirmation

Domains of care were identified from an audit of 1400 children with SB and hydrocephalus over a period of 9 years. Categorically, social function (46%) was the highest domain of care provided, followed by HIV counselling to parents (32%), mobility (16%) and self-care (6%) (Mweshi et al. 2011) The results of the study show levels of how the domains of self-care, mobility and social function were being managed. The facility of HIV counselling to parents was used significantly and hence becomes an important aspect in the whole rehabilitation process of children with SB.

Subsequently, the results of the literature search confirmed the already known three functional domains of self-care, mobility and social function and the two new contributions, being the domains of participation and communication that were identified and included. There is evidence that functional tools have potential to evaluate the impact of clinical interventions (Adolfsson et al. 2010; Bier et al. 2005; Ettling et al. 2006; Ketelaar et al. 2001). Further, functional independence in children can be measured in three areas of self-care, mobility and social cognition using the WeeFIM, PEDI and other measures. It is highly recommended that the ICF-CY-based assessment tool measuring interventions focus on communication and child participation (Adolfsson et al. 2010; Björck-Åkesson et al. 2010; Klang 2012; Morris 2009). Table 4a and Table 4b show the domains that were identified and subsequently confirmed.

**TABLE 4a:** Identified functional domains.

Retrospective study	Literature review	

	Electronic search	Manual search
Social function	Self-care	Self-help skills
HIV counselling	Mobility	Fine motor development
Mobility	Social function	Gross motor development
Self-care	Participation	Interpersonal skills
	Communication	Receptive language development
		Expressive language development

*Source*: Authors’ own work

**TABLE 4b:** Identified functional domains.

Main functional domains	Sub-domains (new domains)
Self-care, mobility, social function	Participation and communication

*Source*: Authors’ own work

### Item generated from qualitative data

Statements generated from interviews of parents and youths were initially pooled and so were those from the focus groups of parents and youths. Eventually, the pooled data from the two different methods were combined to come up with one pool of results leading to a process known as triangulation. Methodological triangulation is the use of two or more different kinds of methods in a single line of inquiry (Risjord 2001). Combinations at the method level can be used to expand the scope of a study as researchers seek to capture method-linked dimensions of a target phenomenon (Greene, Caracelli & Graham 1989). The two methods served as invaluable tools for gathering data, and the benefits were seen from the depths of responses during interviews compared to responses from the focus group. For instance, A9, a male student, had this to share:

*‘*I have no interest in friends because of my smell … they run away.*’*

Another female student, A5 shared:

‘I feel the urge to pass urine, but by the time I reach the toilet, my pants are wet. This makes me always to stay at home.’

The depth of such responses involved pure honesty and such would be quite difficult to share freely for most people. Pooling of items for some researchers is performed during literature search and they just get confirmed during FGDs (Nassar-Mcmillan et al. 2010). The current study opted to pool statements after the interviews and FGD. The statements that were pooled were a homogeneous collection of functional items around the three main domains of self-care, mobility and social function.

The process of selecting items from pools of statements has been practised by several researchers (Babcock-Parziale & Williams 2006; Slaghuis et al. 2011). Selecting items for the current study began first by converting the statements into clear items. For instance, a parent coded B3 during semi-structured interviews shared this:

‘My child cannot feed himself although he is 4 years.’

The deduced item was self-feeding and the functional domain identified was self-care domain. An initial pool of 150 statements enabled the key concepts to be identified and after checking for redundancy, colloquialisms and ambiguity, the number of statements was reduced to 100 statements. Table 5 presents a pooling of statements from both semi-structured interviews and FGDs. These statements were further categorised and thus reduced to 90 items and later categorised into the three main domains of function, being self-care, mobility and social function. Following the conceptual plan of the blueprint, a selection of 78 items was made. Subsequently, 36 items were grouped under self-care, mobility 21 items while social function also had 21 items, shown in Table 6. The items that were generated were subsequently subjected to content validity evaluation.

**TABLE 5:** Pooling of statements from both semi-structured interviews and focus group discussions.

Deduced items (Parents/Caregivers)	Domain code	Deduced items (youths)	Domain code
Choice of food and drink	SC	Bowel opening, cleaning	SC
Hand use and drinking	SC	Bladder and bowel control/friends	SC
Self-feeding	SC	Bladder and bowel control	SC
Feeding	SC	Bladder and bowel control	SC
Hunger and thirst expression	SC	Bladder and bowel control	SC
Brushing teeth	SC	Bladder and bowel control	SC
Dressing and undressing	SC	Dressing and undressing/bathing	SC
Dressing and undressing	SC	Dressing and undressing	SC
Choices/feeding and clothes	SC	Sensation/self-care	SC
Bathing	SC	Sensation/self-care	SC
Bladder and bowel care	SC	Brushing teeth/washing face	SC
Bladder control/not able to relate	SC/SF	Feeding	SC
Bowel opening, cleaning, cooking food, dependency	SC/SF	Choices/feeding	SC
Movement and standing	MO	Hand use	MO
Sitting and moving	MO	Moving	MO
Crawling, sitting and walking	MO	Movement	MO
Walking	MO	Hand use	MO
Hand use	MO	Moving	MO
Moving	MO	Walking	MO
Moving	MO	Talking	SF
Walking	MO	Relationships	SF
Talking	SF	Roles/participation	SF
Playing/relating	SF	Can draw water, cooking food	SF
Responsibility and participation	SF	Helping in daily routine chores/participation	SC/SF
Talking and relationships	SF	Relationships	SF
No interest in people	SF	Playing	SF
Can draw water	SF	Interest in people	SF
Language development/ hearing	SF	Exploring things	SF
Exploring things	SF	Helping in daily routine chores/participation	SF
No friends, stopped schooling	SF	Many friends/schooling	SF
Talking and communication	SF	Talking and communication	SF

*Source*: Authors’ own work

SC, Self-care; MO, Mobility; SF, Social function.

**TABLE 6:** The 78 items identified for the content validity evaluation.

Variables	Self-care (36 items)	Mobility (21 items)	Social function (21 items)
**Feeding**	Thirst expression Choice of drink Opening the mouth Swallowing of fluids Use of hands in drinking Preparing for a drink Hunger expression Choice of food Self-feeding of soft foods Use of utensils when eating Chewing solid foods Serve food		
**Transfers**		Changing positions in bed From lying to sitting From sitting to standing From standing to sitting down on the floor From sitting down on the floor to kneeling From kneeling to sitting From kneeling to standing	
**Use of hands**		Bilateral use of hands Unilateral use of hand Lifting objects up Fine use of hands	
**Communication**			Hearing Response Language development Time orientation Self-information Vocabulary development Expression Conversation
**Social interaction**			Interpersonal relationships Family relationships Informal relationships Interest in exploring new things Playing by self Playing with objects Playing with adults Playing with peers
**Toileting**	Communicating the urge to pass urine Removal of pants before passing urine Change pants in cases of messing up Communicating the urge to open bowels Going to the toilet to open bowels Sitting/squatting on a toilet/potty Cleaning self after opening the bowels Times of opening bowels in a day		
**Locomotion**		Walking or crawling/shuffling on a flat surface Walking or crawling/shuffling up stairs Walking or crawling/shuffling down stairs Moving within the home buildings Moving within buildings other than the home Moving outside buildings Carrying objects while moving Picks up objects Jumping Running	
**Social responsibility**			Undertaking a single task Undertaking multiple tasks in the home Undertaking multiple tasks outside the home Undertaking daily routine Going to preschool
**Bathing and grooming**	Sitting with balance during bathing Standing with balance during bathing Brushing teeth Washing face		
**Dressing and undressing**	Choice of clothes Wearing pants and shorts/skirt Wearing of shirt or dress Putting on socks Putting on shoes Taking off the shirt or dress Taking off shoes Taking off socks Taking off shorts/skirt and pants		
**Skin care**	Responding to touch in the lower limbs Responding to pain in the lower limbs Communicating the presence of pressure sores		

*Source*: Authors’ own work

### Content validation

The frequencies of the ratings for the content validity results by the 12 expert specialists are evident in Table 7. Only two items had average ratings less than 3 (somewhat relevant), viz. item 2 ‘Choice of drink’ ($\bar{X}$ = 2.92; s.d. = 0.90) and item 25 ‘Choice of clothes’ ($\bar{X}$ = 2.83; s.d. = 0.72). All other items had average ratings from the 12 expert specialists of 3 (quite relevant) and above, while 10 items received average ratings of 4 (very relevant) indicating that all 12 expert specialists rated these items as very relevant. The average expert specialist rating for all 78 items was 3.78. Table 7 shows the frequencies of ratings by the 12 expert specialists for each item that was employed for the determination of content validity of the measure.

**TABLE 7:** Frequencies of ratings by the expert clinicians and Item Content Validity Indices.

No.	Items	Expert reviewer												Mean	s.d.	I-CVI

		1	2	3	4	5	6	7	8	9	10	11	12			

		PT	PT	PT	*N*	*N*	*N*	CO	PT	PT	CO	MO	MO			
**Self-care**																
	**Feeding**															
1	Thirst expression	3	4	4	4	2	3	4	4	4	4	4	2	3.50	0.80	0.83
2	Choice of drink	4	3	4	2	2	4	3	3	3	3	3	1	2.92	0.90	0.75
3	Opening the mouth	4	4	4	4	3	4	4	4	4	4	3	3	3.75	0.45	1.00
4	Swallowing of fluids	4	4	4	4	4	4	4	4	4	4	4	2	3.83	0.58	0.92
5	Use of hands in drinking	4	3	4	4	4	4	4	4	3	4	4	4	3.83	0.39	1.00
6	Preparing for a drink	4	3	4	4	4	4	4	2	4	3	3	1	3.33	0.98	0.83
7	Hunger expression	4	4	4	4	4	4	4	4	4	4	4	2	3.83	0.58	0.92
8	Choice of food	4	4	4	3	4	4	3	3	3	3	3	2	3.33	0.65	0.92
9	Self-feeding of soft foods	4	3	4	4	4	4	4	3	4	4	3	3	3.67	0.49	1.00
10	Use of utensils when eating	4	4	4	3	4	4	4	2	4	4	3	3	3.58	0.67	0.92
11	Chewing solid foods	4	4	4	4	4	4	4	4	4	3	4	3	3.83	0.39	1.00
12	Serve food	3	4	4	2	4	3	4	2	3	4	2	1	3.00	1.04	0.67
	**Toileting**															
13	Communicating the urge to pass urine	4	4	4	4	4	4	4	4	4	4	4	4	4.00	0.00	1.00
14	Removal of pants before passing urine	4	4	4	3	4	4	4	4	4	4	4	3	3.83	0.39	1.00
15	Change pants in cases of messing up	4	4	4	4	4	4	4	4	4	4	4	3	3.92	0.29	1.00
16	Communicating the urge to open bowels	4	4	4	4	4	4	4	4	4	4	4	4	4.00	0.00	1.00
17	Going to the toilet to open bowels	4	4	4	4	4	4	4	4	4	4	4	3	3.92	0.29	1.00
18	Sitting/squatting on a toilet/potty	4	4	4	3	4	4	4	4	4	4	3	4	3.83	0.39	1.00
19	Cleaning self after opening the bowels	4	4	4	3	4	4	4	4	4	3	3	3	3.67	0.49	1.00
20	Times of opening bowels in a day	4	3	4	3	4	4	3	3	3	4	3	2	3.33	0.65	0.92
	**Bathing and grooming**															
21	Sitting with balance during bathing	4	4	4	4	4	4	4	4	4	4	4	3	3.92	0.29	1.00
22	Standing with balance during bathing	4	4	4	4	4	4	4	4	4	4	3	3	3.83	0.39	1.00
23	Brushing teeth	4	4	4	4	4	4	4	3	4	4	3	3	3.75	0.45	1.00
24	Washing face	4	3	4	3	4	4	4	4	4	4	3	2	3.58	0.67	0.92
	**Dressing and undressing**															
25	Choice of clothes	4	3	4	2	2	3	3	2	3	3	3	2	2.83	0.72	0.67
26	Wearing pants and shorts/skirt	4	4	4	4	4	4	4	3	4	4	4	2	3.75	0.62	0.92
27	Wearing of shirt or dress	4	4	4	4	4	4	4	4	4	4	4	2	3.83	0.58	0.92
28	Putting on socks	4	3	4	3	4	4	4	4	3	4	4	3	3.67	0.49	1.00
29	Putting on shoes	4	4	4	3	4	4	4	4	3	4	4	3	3.75	0.45	1.00
30	Taking off shirt or dress	4	4	4	4	4	4	4	4	4	4	4	3	3.92	0.29	1.00
31	Taking off shoes	4	4	4	4	4	4	4	4	3	4	4	3	3.83	0.39	1.00
32	Taking off socks	4	4	4	4	4	4	4	4	3	4	4	3	3.83	0.39	1.00
33	Taking off shorts/skirt and pants	4	4	4	4	4	4	4	4	4	4	4	3	3.92	0.29	1.00
	**Skin care**															
34	Responding to touch in the lower limbs	4	4	4	4	4	4	4	4	4	4	3	4	3.92	0.29	1.00
35	Responding to pain in the lower limbs	4	4	4	4	4	4	4	4	4	4	4	4	4.00	0.00	1.00
36	Communicating the presence of pressure sores	4	4	4	4	4	4	4	4	4	4	4	4	4.00	0.00	1.00
**Mobility**																
	**Transfers**															
37	Changing positions in bed	4	4	4	4	4	4	4	4	4	4	4	4	4.00	0.00	1.00
38	From lying to sitting	4	4	4	4	4	4	4	3	4	4	4	4	3.92	0.29	1.00
39	From sitting to standing	4	4	4	4	4	4	4	3	4	4	4	3	3.83	0.39	1.00
40	From standing to sitting down on the floor	4	4	4	4	4	4	4	3	4	4	4	3	3.83	0.39	1.00
41	From sitting down on the floor to kneeling	4	4	4	4	4	4	4	3	4	4	3	3	3.75	0.45	1.00
42	From kneeling to sitting	4	4	4	4	4	4	4	3	4	4	3	3	3.75	0.45	1.00
43	From kneeling to standing	4	4	4	4	4	4	4	3	4	4	3	3	3.75	0.45	1.00
	**Use of hands**															
44	Bilateral use of hands	4	4	4	4	4	4	4	4	4	4	4	4	4.00	0.00	1.00
45	Unilateral use of hand	4	4	4	4	4	4	4	3	4	4	4	3	3.83	0.39	1.00
46	Lifting objects up	4	4	4	4	4	3	4	4	4	4	3	3	3.75	0.45	1.00
47	Fine use of hands	4	4	4	4	4	4	4	4	4	4	4	3	3.92	0.29	1.00
	**Locomotion**															
48	Walking or crawling/shuffling on a flat surface	4	4	4	4	4	4	4	3	4	4	4	4	3.92	0.29	1.00
49	Walking or crawling/shuffling up stairs	4	4	4	4	4	4	4	3	4	4	3	4	3.83	0.39	1.00
50	Walking or crawling/shuffling down stairs	4	4	4	4	4	4	4	3	4	3	3	4	3.75	0.45	1.00
51	Moving within the home buildings	4	4	4	4	4	4	4	3	4	4	4	4	3.92	0.29	1.00
52	Moving within buildings other than the home	4	4	4	4	4	4	4	3	4	4	3	3	3.75	0.45	1.00
53	Moving outside buildings	4	4	4	4	4	4	4	4	4	3	3	3	3.75	0.45	1.00
54	Carrying objects while moving	4	4	4	4	4	4	4	3	4	2	3	3	3.58	0.67	0.92
55	Picks up objects	4	4	4	4	4	4	4	4	4	4	4	3	3.92	0.29	1.00
56	Jumping	4	4	4	4	4	4	4	3	4	4	3	2	3.67	0.65	0.92
57	Running	4	4	4	4	4	4	4	3	3	4	3	2	3.58	0.67	0.92
**Social function**																
	**Communication**															
58	Hearing	4	4	4	4	4	4	4	4	4	4	4	4	4.00	0.00	1.00
59	Response	4	4	4	4	4	4	4	4	4	4	4	3	3.92	0.29	1.00
60	Language development	4	4	4	4	4	4	4	4	4	4	4	4	4.00	0.00	1.00
61	Time orientation	4	4	4	4	4	4	4	3	4	4	4	4	3.92	0.29	1.00
62	Self-information	4	4	4	4	4	4	4	3	4	4	4	3	3.83	0.39	1.00
63	Vocabulary development	4	4	4	4	4	4	4	4	4	4	4	3	3.92	0.29	1.00
64	Expression	4	4	4	4	4	4	4	4	4	4	4	3	3.92	0.29	1.00
65	Conversation	4	4	4	4	4	4	4	4	4	4	4	3	3.92	0.29	1.00
	**Social interaction**															
66	Interpersonal relationship	4	4	4	4	4	4	4	4	4	4	4	3	3.92	0.29	1.00
67	Family relationships	4	4	4	4	4	4	4	4	4	4	4	4	4.00	0.00	1.00
68	Informal relationships	4	4	4	4	4	4	4	4	4	4	3	3	3.83	0.39	1.00
69	Interest in exploring new things	4	4	4	4	4	4	4	4	4	4	4	3	3.92	0.29	1.00
70	Playing by self	4	4	4	4	4	4	4	4	4	3	3	3	3.75	0.45	1.00
71	Playing with objects	4	4	4	4	4	4	4	4	4	4	3	3	3.83	0.39	1.00
72	Playing with adults	4	4	4	4	4	4	4	3	4	3	3	3	3.67	0.49	1.00
73	Playing with peers	4	4	4	4	4	4	4	4	4	4	4	4	4.00	0.00	1.00
	**Social responsibility**															
74	Undertaking a single task	4	4	4	4	4	4	4	4	4	4	4	3	3.92	0.29	1.00
75	Undertaking multiple tasks in the home	4	4	4	4	4	4	4	4	4	4	4	3	3.92	0.29	1.00
76	Undertaking multiple tasks outside the home	4	4	4	4	4	4	4	4	4	4	3	3	3.83	0.39	1.00
77	Undertaking daily routine	4	4	4	4	4	4	4	3	4	4	4	3	3.83	0.39	1.00
78	Going to pre-school	4	4	4	4	4	4	4	4	3	4	4	4	3.92	0.29	1.00

PT, Physiotherapy; N, Nurse; CO, Clinical Officer; MO, Medical Officer; I-CVI, Item Content Validity Indices.

I-CVIs were calculated for each item by counting the number of experts who rated the items as either somewhat relevant (3) or very relevant (4) and then dividing that total by the number of expert specialists (Polit & Beck 2006). As mentioned above, items 2 (Choice of drink) and 25 (Choice of clothes) had the lowest average ratings and thus the lowest I-CVI scores (0.75 and 0.67 respectively). In addition, item 12 (Serve food) also had an I-CVI of 0.67. Two items, item 1 (Thirst expression) and item 6 (Preparing for a drink) had I-CVIs of 0.83, while 11 items had I-CVIs of 0.92. The remainder of the items (62 items) had I-CVIs of 1.00 indicating that all 12 expert specialists considered these items as either somewhat relevant or very relevant. Polit and Beck (2006) argue that S-CVIs can be calculated by dividing the number of items that all experts considered either somewhat or very relevant by the total number of items. In this instance our 62 items divided by the total items (78) result in an S-CVI of 0.80, which is the standard criterion for acceptability (Polit & Beck 2006).

When the 78 items were exposed to reliability analysis, the alpha coefficient of 0.98 was computed. When the items were further analysed in categories of the three main domains, the results showed that the alpha coefficient for self-care was 0.97, mobility was 0.95 and social function had an alpha of 0.95. Therefore, results for both the CVI and alpha coefficient were above 0.80, indicating an acceptable level of content validity (Waltz et al. 2010).

Based on such results, decisions had to be made on the following three items:

- choice of drink (self-care)
- choice of clothes (self-care)
- serve food (self-care).

It was recommended that item 12 (Serve food) under self-care domain be removed thus reducing the number of items to 77. However, items 2 (Choice of drink) and 25 (Choice of clothes) were recommended for reliability evaluation.

### Instrument construction: Zambia Spina Bifida Functional Measure

The measure with 77 items was finally assembled including the preparation of the cover page with important information, directions, scoring keys and answer sheets. Subsequent to compiling all important documents, the first draft of the tool, titled ‘Zambia Spina Bifida Functional Measure’ (ZSBFM), designed for evaluating the performance of functional skills in children with SB in Zambia, was developed.

The ZSBFM is aimed at measuring the impact of interventions like surgery and physiotherapy given to children with SB from the age of 6 months to 5 years. The ZSBFM draft had two sections: Section A: demographic data, while Section B: 77 items categorised in three domains of self-care, mobility and social function. From the 77 items, 37 (48%) were under the self-care domain, 19 (25%) mobility domain and 21 (27%) under the social function domain.

## Discussion

Faced with the clinical problem of lack of evidence on the impact of interventions given to children with SB, the researchers set out to develop a tool expected to fill the gap that existed. The intent was to locally generate a measure with psychometric adequacy that could readily be available, affordable, appropriate and culturally sensitive in assessing the performance of functional skills in children with SB in Zambia. A retrospective study was conducted to identify domains and through a systematic review, the domains were confirmed. Subsequently, items were generated, content validation was performed, and subsequently the first draft was constructed.

Domains of care identified from the retrospective study showed that social function was the highest care provided, followed by HIV counselling to parents, mobility and self-care. Mobility performed fairly in the management of children with SB in Zambia. Although mobility performed fairly, such impairments are very common among individuals with SB (Haley et al. 2010; Jahnsen et al. 2002) and many lead sedentary lives compared to those without disabilities (Willemsen & Fons 1997). The problem of mobility can be quite overwhelming in Zambia where accessibility for Persons with Disabilities is quite a big challenge. Despite mobility limitations in some individuals with SB, a lot is expected from them by society for them to be accepted and appreciated. This can be confirmed by a study that was conducted in Zambia which revealed that boys are involved in gardening, fetching firewood, running errands and washing plates. The chores for girls include washing plates, fetching water and firewood, bathing babies, running errands, pounding food and cooking (Chibuye et al. 1986). Such demands must be taken into consideration when carrying out interventions for individuals with SB. Perhaps this should motivate clinicians to look for ways and means of rehabilitating individuals with mobility problems in order to prepare them participate in chores expected of them regardless of their physical status.

Even though self-care was rated poorly in terms of care given to children with SB in the current study, literature reveals that only about half of children with SB are able to live independently and almost a quarter of them experience both urinary and faecal incontinence in their lives (Adeleye et al. 2010; Blenchowe et al. 2010). In spite of the global problems of self-care with problems of the bladder and bowel, Zambian children may have different demands considering the cultural variations and implications. For instance, Zambian children are expected to begin eating on their own at a young age (Evans & Myer 1994) considering that most mothers just abruptly stop breastfeeding which could affect a growing child negatively if not observed carefully.

Participation and communication were identified through a systematic review as new sub-domains recommended by the ICF-CY (Klang 2012). It must be noted that the two sub-domains were not measured in earlier developed measures such as the PEDI and WeeFIM, but these have been identified as important domains. These domains may include for example, mobility, self-care, participation, communication, social relationships, leisure or play, education, domestic chores and community integration (Morris 2009). One of the critical issues rehabilitation professionals need to address is how physiotherapeutic exercises or other clinical interventions given to a child with disabilities can be measured using functional outcomes tools. The results of the clinical trial study of Ketelaar et al. (2001) show that the task-specific approach is more effective than the one that takes into account the motor function in a developmental manner. Additionally, the task-oriented approach has proven to be a systematic way of trying to solve a child’s functional problems. The current knowledge that has been gained in the use of the ICF-CY has come with other measurement challenges such as the inclusion of participation and communication in new measures as presented by Adolfsson and colleagues and Morris and colleague (Adolfsson 2011; Morris 2009). The new challenge is calling on rehabilitation professionals such as physiotherapists to plan the task-oriented functional approaches in such a way that they become inclusive of participatory tasks which are also age-oriented by nature.

The process of item generation involved the use of two methods, being semi-structured interviews and FGDs. Several simultaneous steps have been reported in the process of item generation, which eventually led to a pool of items based on a thorough literature review, existing scales, expert opinion (Delamere, Wankel & Hinch 2001) and eventually leading to FGDs (Nassar-Mcmillan et al. 2010). The researchers of the current study utilised the reported several steps except that instead of involving expert opinion in the beginning, a retrospective study was performed to evaluate and identify important functional domains and eventually semi-structured interviews and FGDs were conducted. Some studies (Delamere et al. 2001; Saldana 2009) have used focus groups to confirm the items and also identify domains, whereas the current study used already confirmed domains from a systematic review study and eventually used them to generate items using semi-structured interviews and FGDs. Selecting appropriate data-recording strategies that would help organise data is recommended (Saldana 2009). Of paramount importance to data quality is the accuracy of the transcribed interviews and FGD notes (Waltz et al. 2010). Given the purpose of the study and the type of data collected, the choice of type of analysis was content analysis. Because the researchers wanted to capture the experiences and views of parents or caregivers of children with SB and youths with SB concerning functional skills, they opted to transcribe and present data in verbatim form. Data were then placed into categories of the main domains of care deductively. The results of the two methodologies were categorised under similar themes and finally the back and forth potential verification with some of the original information helped to strengthen the analysis.

The concept of validity refers to the degree to which an instrument measures what it is supposed to measure (Dekker, Dallmeyer & Lankhorst 2005). The procedures for validity evaluation of the current study focused on content. Criterion validity was not included in the plan because a gold standard is frequently not available in rehabilitation, which precludes evaluation of criterion validity (i.e. the degree to which the scores on an instrument correspond to the scores on the gold standard). The process of content-relevant evidence in the current study included the initial restricting of item selection to the test blueprint and obtaining content validity ratings from subject matter experts. Content validity is often viewed as the minimum psychometric requirement for measurement adequacy and is the first step in construct validation of a new measure. It must be built into the measure through the development of items (Waltz et al. 2010). A sample of 12 clinicians was identified comprising 5 physiotherapists, 3 neuro-nurses, 2 clinical officers, 1 neuro-pediatrician and 1 neurosurgeon. The professionals’ average years of experience in child health services was 22 years (s.d. = 8.82) and had academic qualifications in their respective fields (MSc, BSc and Diploma). It is suggested that a minimum of five experts in the field are recommended to judge the content domains of an instrument (Dempsey & Dempsey 1986). The sample size identified for the study was quite adequate, and there was a good and wide representation considering the team of clinicians who manage children with SB in Zambia. The level of agreement between the 12 experts was determined via coefficient alpha in order to measure the content validity. Item as well as S-CVIs were calculated and were indicative of a validity of the measure by separately evaluating each item (Waltz et al. 2010). Additionally, the alpha coefficient computed for the scale items that were generated was between 0.95 and 0.98, showing an acceptable level of content validity (Martuza 1977). Given the good internal consistency as well as the good I-CVIs and S-CVI, we argue that the ZSBFM for children with SB in Zambia is contextually relevant and valid for use in this context.

## Conclusion

A draft measure titled ZSBFM for children with SB in Zambia has been developed. It is meant to help clinicians managing children with SB measure the impact of interventions such as surgery and physiotherapy given to children aged 6 months to 5 years. The measure can provide an opportunity to assess children with SB in performing distinct functional skills based on 77 items categorised into the three main domains of self-care, mobility and social function. The draft ZSBFM has an acceptable level of content validity. Psychometric properties of reliability and validity were measured through Cronbach’s alpha reliability and later I-CVIs and S-CVIs.

## Appendix 1

BOX 1-A1 Summary of questions used in the semi-structured interviews and focus group discussions.

**Table:**

**Questions asked to parents/caregivers of children with SB**	
-How has been the experience of taking care of your child? -Can you share the experience of feeding your child? -Can your child suck milk properly? Can your child chew soft foods? -Can your child swallow food properly? Is your child able to feed itself?	-What happens when your child wants to eat or drink something? Can she/he express the need for something to eat or drink? -Can she/he feed herself? Can your child sit during feeding and bathing? Can she/he dress and undress himself?
-How is toileting done? -Can your child show the need to go to pass urine? Open the bowel? -Can your child brush her/his teeth? -Can she manage her/his shoes? Socks? -Can your child talk? Can your child communicate? -Can your child pick up objects? -How is the activity in the hands? Can your child play with the hands? Can your child lift up objects?	-How is the movement in bed? Can your child turn in bed? Change positions in bed? -How is the mobility in your child out of bed? Can your child crawl? Stand, walk, run? -Can your child carry small things when crawling, walking? -Is your child able to move in the home and outside the home? -How is the balance in sitting? When bathing? When eating?
-Does your child go to school? -Does your child go to church? -Can your child play alone, with peers? -Can your child play with adults? -Does your child participate in home activities?	-Does your child recognise her/his name? respond to stimulus? -How does your child relate with the family members and other people? -Can your child recognise familiar faces? -How is the sense of hearing in your child?
**Questions asked to youths with SB**	
-How independent are you? -How do you manage your bladder and bowel activities? -How many times do you open the bowels in a day? -What do you use for protection if you cannot control your bladder and bowel? -Do you manage to clean yourself after using the toilet?	-Can you manage to prepare water for a bath if you have no tap water? -Can you dress yourself and undress? -Who manages your shoes? Socks? -Can you button up your shirt?
-What are your responsibilities? -How much do you participate in activities of the home like cooking, sweeping and drawing water? -Do you participate in any activities outside the home? If so, what activities? -What roles do you play at home? -How is the laundry done? -Who washes and irons your clothes? -How entrusted are you in family chores? -Can you go to the shops to buy things?	-How is the sensation in the legs? -Can you feel when you touch your feet? -Do you manage to check your skin in your feet? -Can you sweep your bedroom? Other rooms in the house? -Can you go visiting alone? -How involved are you in food preparation? -Can you make your own food? -Can you help prepare food for the family
-How mobile are you and how do you function in the home? -How is your relationship with family members and non-family members? Do you have friends? Do you visit them? -Do they visit you? What games do you enjoy watching? Do you play any games at all? What games do you like playing?	-Do you go to school? -If in school, how is your experience? -Have you been to college? -If so, how was your experience in college?
**Questions discussed with caregivers of children with SB**	
-Can we discuss experiences of taking care of our children -How has been the experience of feeding the children? -Can your child express the desire to eat and drink? -How can you tell that your child wants something to eat or drink?	-Let us discuss issues of movement: -Can one share how her child moves in the house from one place to another? -How is the experience of standing, walking and running?
-Can we talk about movements the children can do: -Is there any form of activity in the arms? -Can anything be lifted? -Can your child touch its mouth? -Is there any movement in bed? -Can your child come to sitting from lying in bed?	-Let us talk about communication and relationships -How does your child relate with people around and those who visit in the home? -Can your child talk? -Can your child communicate the need to eat, drink, go to the toilet or even the need to play or others?
-Let us talk about participation of our children: -What activities does your child do to participate in the activities of the home? -Does your child participate in home activities? Bathing experience? -Can your child brush her/his teeth? -Can she/he dress himself?	-Let us talk about personal care: -Does the child play with others? -Can your child feel when you touch the legs and feet? -Does your child go to school? -Can she/he manage her/his shoes? -Can she/he show the need to go to pass urine?
**Questions discussed with youths with SB**	
-Can we discuss our personal experience of independence -How independent are we in feeding? -Can you express what you desire to eat and drink?	-Let us discuss issues of movement: -How mobile are you? Can anyone share the experience of moving in the house from one place to another? How are the standing, walking and running?
-Can we talk about your movements: -Is there any form of activity in the arms? -Can anything be lifted? -Can you touch the mouth? -How is the movement in bed? -Can you come to sitting from lying in bed?	-Let us talk about communication and relationships -How easy is it for you to communicate? -How do you relate with people around and those who visit in the home? -Can you communicate the need to eat, drink, go to the toilet or even the need to do other things?
-Let us talk about your participation: -What activities do you participate in? Do you participate in home activities? Bathing experience? Can you cook? -Can you brush your teeth? -Can you dress yourself?	-Let us talk about personal care: -Are you able to take care of your personal needs? -Can you manage your shoes? -Can you tell the need to go to pass urine? -Can you feel when you touch the legs and feet? -Do you go to school or college?
